# Effects of Coenzyme Q10 on Markers of Inflammation: A Systematic Review and Meta-Analysis

**DOI:** 10.1371/journal.pone.0170172

**Published:** 2017-01-26

**Authors:** Junya Zhai, Yacong Bo, Yan Lu, Chunli Liu, Lishi Zhang

**Affiliations:** 1 Department of Nutrition, Food Safety and Toxicology, West China School of Public Health, Sichuan University. Chengdu, China; 2 Department of Nutrition and Food Hygiene, College of Public Health, Zhengzhou University, Zhengzhou, Henan, China; 3 Department of Surgery, Shanghai Ninth People’s Hospital (North), Shanghai Jiaotong University School of Medicine, Shanghai, China; 4 The First Affiliated Hospital of Zhengzhou University, Zhengzhou, Henan, China; Universidad Pablo de Olavide, SPAIN

## Abstract

**Background/Objective:**

Chronic inflammation contributes to the onset and development of metabolic diseases. Clinical evidence has suggested that coenzyme Q10 (CoQ10) has some effects on inflammatory markers. However, these results are equivocal. The aim of this systematic review was to assess the effects of CoQ10 on serum levels of inflammatory markers in people with metabolic diseases.

**Methods:**

Electronic databases were searched up to February 2016 for randomized controlled trials (RCTs). The outcome parameters were related to inflammatory factors, including interleukin-6 (IL-6), tumor necrosis factor-alpha (TNF-α) and C reactive protein (CRP). RevMan software was used for meta-analysis. Meta-regression analysis, Egger line regression test and Begg rank correlation test were performed by STATA software.

**Results:**

Nine trials involving 428 subjects were included in this meta-analysis. The results showed that compared with control group, CoQ10 supplementation has significantly improved the serum level of CoQ10 by 1.17μg/ml [MD = 1.17, *95% CI* (0.47 to 1.87) μg/ml, *I*^2^ = 94%]. Meanwhile, it has significantly decreased TNF-α by 0.45 pg/ml [MD = -0.45, *95% CI* (-0.67 to -0.24) pg/ml, *I*^2^ = 0%]. No significant difference was observed between CoQ10 and placebo with regard to CRP [MD = -0.21, *95% CI* (-0.60 to 0.17) mg/L, *I*^2^ = 21%] and IL-6 [MD = -0.89, *95% CI* (-1.95 to 0.16) pg/ml, *I*^2^ = 84%].

**Conclusions:**

CoQ10 supplementation may partly improve the process of inflammatory state. The effects of CoQ10 on inflammation should be further investigated by conducting larger sample size and well-defined trials of long enough duration.

## 1. Introduction

Chronic, low-grade systematic inflammation is a common pathogenetic denominator in many diseases. The elevated pro-inflammatory factors, such as C reactive protein (CRP), tumor necrosis factor-alpha (TNF-α), interleukin-6 (IL-6)[1], play a central role in metabolic diseases, including obesity, type 2 diabetes (T2DM), metabolic syndrome (MS), cardiovascular disease[2,3] (CVD) and nonalcoholic fatty liver disease (NAFLD)[4]. Anti-inflammatory agents, old drugs (salicylates, especially salsalate) and new compounds (essentially monoclonal antibodies), are emerging in clinical management of the related chronic inflammatory diseases but the observed improvements appear rather modest, and questions have been remained in poorly known long-term safety and high cost [5]. Therefore, it is essential to find natural compounds for substitutions.

Coenzyme Q10 (CoQ10, ubiquinone-10) is an endogenously synthesized compound that acts as an essential cofactor in the mitochondrial electron transport system, a potent antioxidant of lipid membranes[6] and a modulator of the gene expression[7]. These functions underlie the rationale for its use in clinical practice and food supplement. Previous study reported CoQ10 tends to decrease hepatic mRNA expression of IL-6 and TNF-α[8], to attenuate the level of CRP[9]. Several randomized controlled trials (RCTs) demonstrated the efficacy of CoQ10 as an adjuvant therapeutic in metabolic diseases. However, the results of these trials are inconsistent, because of small sample sizes and uneven quality. Therefore, a meta-analysis of RCTs was conducted here to explore whether the supplementation of CoQ10 exhibits anti-inflammatory benefits.

## 2. Materials and Methods

The meta-analysis was conducted according to the Preferred Reporting Items for Systematic Reviews and Meta-analyses (PRISMA) Guidelines (S1 Checklist).

### 2.1 Search strategy

Systematic search was conducted in the PubMed, MEDLINE, Web of Science, Cochrane Library Databases (up to February 2016) for identifying eligible studies. The following combination of search terms was used in databases in English: (Coenzyme Q10 OR CoQ10 OR ubiquinone-10) AND (inflammation OR system inflammation OR IL-6 OR interleukin-6, OR CRP OR C reactive protein OR high sensitive CRP OR hs-CRP OR TNF-α OR tumor necrosis factor).

### 2.2 Data selection

All titles and abstracts were independently screened by 2 investigators (JZ, YB) to evaluate eligibility for inclusion until consensus was reached. For a study to be included in the systematic review, it had to be: (1) RCTs; (2) participants aged 18 years or older; (3) intervention with capsule of CoQ10; (4) having a control group with placebo; (5) minimum intervention period of four weeks; (6) assessment markers of inflammation (CRP, IL-6, TNF-α); (7) data with normal distribution and report of mean and standard deviation (SD) or available data (standard errors, 95% confidence interval, *p*-values) to calculate these values.

### 2.3 Data extraction

Data extraction was performed independently by two authors (JZ, YB). The data recorded were publication year, daily dose of supplemental CoQ10, study design, intervention duration, country, design details, health status, age and number of participants, side- effects, means and SD data of each parameter before and after the intervention.

### 2.4 Assessment of risk of bias

The risk of bias (high risk, low risk or unclear risk of bias) was assessed independently with the Cochrane Hand book for Systematic Reviews of Interventions by two reviewers (JZ, CL) for each study[10]. The following methodological domains were considered: random sequence generation, allocation concealment, blinding of participants and personnel, blinding of outcome assessment, incomplete outcome data, selective reporting, and other potential threats to validity.

### 2.5 Data analysis

Pooled effect size was expressed as weighted mean differences (MD) and corresponding *95% CI* for each parameter in this meta-analysis. The heterogeneity of the included studies was examined by *χ*^2^ tests and the degree of heterogeneity was estimated using *I*^2^ statistic. The fixed-effect model (*I*^*2*^ was below 50%)[11] or the random-effects model (*I*^*2*^ was above 50%)[12] was chosen for meta-analysis of the comparison of each parameter due to CoQ10 treatment compared to that due to placebo. Meta-analysis was performed to determine potential effect modification of variables including age, BMI, dose of CoQ10 supplementation, intervention duration, sample size and the baseline concentration of each parameter. Potential publication bias was explored by using funnel plot, Egger line regression test (Egger’s test) [13] and Begg rank correlation test (Begg’s test) [14]. RevMan software (version 5.2) was used for meta-analysis. Meta-regression analysis, Egger’s test and Begg’s test were performed by STATA software (version 12.0; StatCorp, College Station, TX, USA). *P*< 0.05 was considered as statistically significant. All statistical tests were two-sided.

## 3. Results

### 3.1 Description of studies

**Fig 1:**
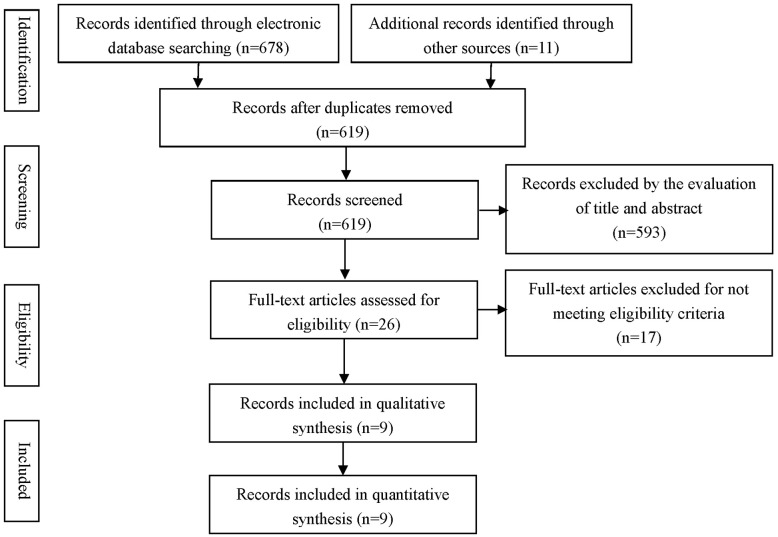
Flow diagram of the study selection process.

The initial search identified 619 articles, of which 593 were excluded based on their titles and abstracts. After a full-text review of the remaining 26 potentially relevant articles, 17 articles were further excluded because 4 articles used compound agents other than CoQ10, 6 articles measured an acute effect, 2 articles did not meet a specific RCT design, 2 articles showed incomplete data, 2 articles had non-normally distributed variables and the other one did not provide the exact number of subjects in each intervention and control groups. Finally, Nine papers with RCTs were selected for meta-analysis [15–23] (Fig 1).

### 3.2 Study characteristics

The characteristics of the included trials were presented in Table 1. Nine trials involving 428 subjects were published from 2009 to 2015, in which five were conducted in Iran[18,19,21–23], two in China[17,20], and the other two in Australia[15] and Korea[16], respectively. A parallel, double blinded design was used in all trials [15–23], which involved patients with metabolic diseases, including obesity, metabolic syndrome, T2DM, CVD, and NAFLD. The intervention duration lasted from eight to twelve weeks. The daily dose of CoQ10 varied from 100 to 500 mg. All control groups received placebo. Of the nine included studies, three reported plasma CoQ10[16,17,20], TNF-α[18–20], respectively, and four for IL-6[18–21], seven for CRP (three for CRP[15,16,20], four for hs-CRP[17,18,22,23]).

**Table 1:** The characteristics of included studies in this study.

Study, year		Mori, 2009 [15]	Lee, 2011 [16]	Dai, 2011 [17]	Farsi, 2015 [18]	Sanoobar, 2015 [19]	Lee, 2013 [20]	Mohseni, 2015 [21]	Raygan, 2015 [22]	Nesami, 2015 [23]
Country		Australia	Korea	China	Iran	Iran	China	Iran	Iran	Iran
Duration (weeks)		8	12	8	12	12	12	12	8	12
Coenzyme Q10 dose (mg/d)		200	200	300	100	500	300	200	100	100
Health status		risk of CVD in chronic kidney disease	obesity	coronary artery disease	NAFLD	multiple sclerosis	coronary artery disease	myocardial infarction	metabolic syndrome	hypertension
Adverse effects		NR	NR	No	redness, itchiness	NR	No	NR	No	No
Age, years	CoQ10	55.4±2.7^a^	42.7±11.3^a^	67.7±9.4^a^	19–54^b^	33.1± 7.6^a^	71.7±11.5^a^	60.0±8.0^a^	65.9±12.5^a^	49.7±5.65^a^
	Placebo	58.6±2.6^a^	42.5±11.2^a^	70.1±9.8^a^	19–54^b^	30.9± 7.7^a^	66.5±11.1^a^	61.0±7.0^a^	59.9±13.1^a^	48.07±6.33^a^
Sample size	CoQ10	21	17	28	20	22	23	26	30	30
	Placebo	15	19	28	21	23	19	26	30	30
CoQ10,μg/mL (Mean±SD)	CoQ10	NR	0.58±0.24	1.08±0.41	NR	NR	NR*	NR	NR	NR
	Placebo	NR	0.65±0.27	0.95±0.29	NR	NR	NR*	NR	NR	NR
IL-6, pg/mL (Mean±SD)	CoQ10	NR	NR	NR	2.15± 0.98	1.52± 2.4	NR*	17.7±7.65	NR	NR
	Placebo	NR	NR	NR	2.19 ± 0.9	1.53± 2.2	NR*	12.61±5.91	NR	NR
TNF-α, pg/mL (Mean±SD)	CoQ10	NR	NR	NR	1.38 ± 0.71	6.2 ±0.97	NR*	NR	NR	NR
	Placebo	NR	NR	NR	1.27± 0.09	6.09±0.92	NR*	NR	NR	NR
CRP,mg/L (Mean±SD)	CoQ10	1.46±2.87	1.42±1.13	2.29±3.10	18.81 ± 6.96	NR	NR*	NR	1.51±1.57	3.53 ± 3.36
	Placebo	1.56±2.87	1.04±1.45	1.48±1.82	19.85 ± 9.14	NR	NR*	NR	2.91±3.05	4.12 ± 3.20

*Note*: CVD, cardiovascular disease; NAFLD: nonalcoholic fatty liver disease; CoQ10: Coenzyme Q10; IL-6: interleukin-6; TNF-α:tumor necrosis factor-alpha, CRP: C reactive protein; NR, not reported.

*the original basic data was not provided and the mean change was replaced;

^a^, described as Mean±SD;

^b^, described as minimums and maximums.

### 3.3 Risk of bias in the included studies

**Fig 2:**
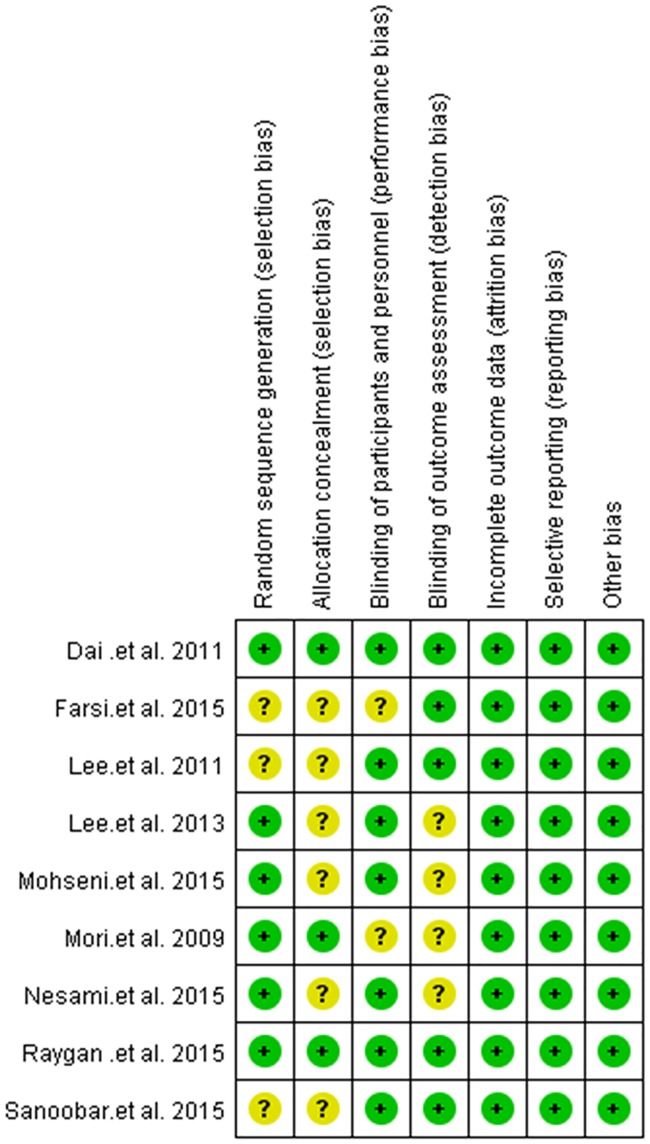
Risk of bias assessment of included studies.

The assessment of risk of bias was presented in Fig 2. All studies were randomized trial design, while three trials had[16,18,19] no detailed information about random sequence generation and six studies[16,18–21,23] did not describe the methods of allocation concealment. Blinding of participants, personnel and outcome assessment were considered to be unclear in two trials [15,18] and four trials [15,20,21,23], respectively.

### 3.4 Effects of interventions

**Fig 3:**
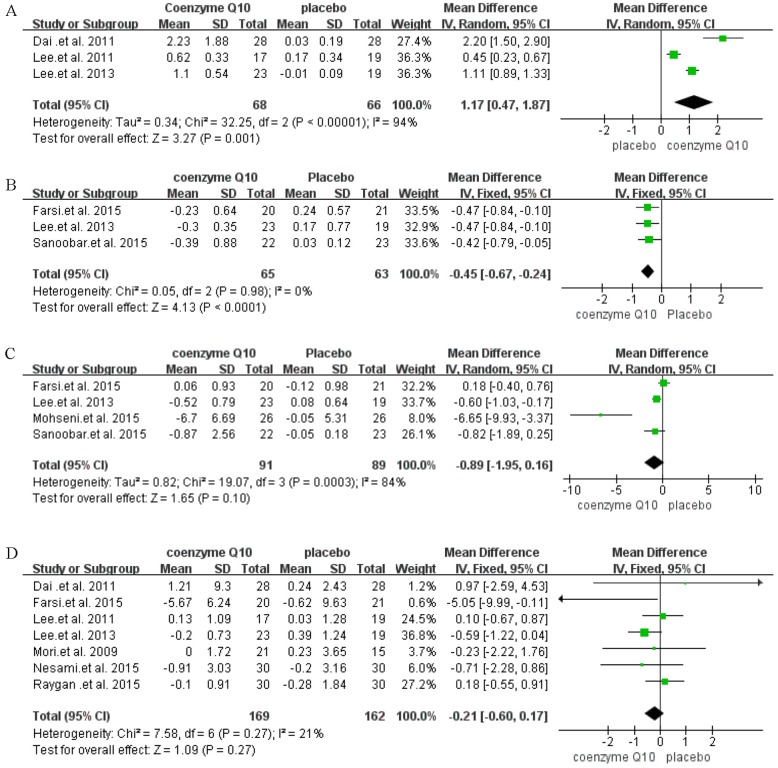
Forest plot of comparisons of Coenzyme Q10 supplementation versus placebo (outcomes: A: serum Coenzyme Q10, B: tumor necrosis factor-alpha, C: interleukin-6, D: C reactive protein).

The effects of CoQ10 supplementation on the levels of plasma CoQ10, TNF-α, IL-6 and CRP are shown in Fig 3. The pooled analysis showed that the CoQ10 supplementation group had higher levels of CoQ10 [MD = 1.17, *95% CI* (0.47 to 1.87) μg/ml, *I*^*2*^ = 94%] (Fig 3A) than the control group. Meanwhile, CoQ10 significantly decreased TNF-α level [MD = -0.45, *95% CI* (-0.67 to -0.24) pg/ml, *I*^2^ = 0%] (Fig 3B) compared with that of the control group. However, no statistically significant difference was found in the levels of IL-6 [MD = -0.89, *95% CI* (-1.95 to 0.16) pg/ml, *I*^2^ = 84%] (Fig 3C) and CRP [MD = -0.21, 95% *CI* (-0.60 to 0.17) mg/L, *I*^2^ = 21%] (Fig 3D).

### 3.5 Heterogeneity analysis

A univariate meta-regression analysis was conducted to explore the sources of heterogeneity by using age, BMI, dose of CoQ10 supplementation, intervention duration, and sample size and baseline concentration of each parameter. None of these covariates influenced the pooled effect significantly.

### 3.6 Publication bias

Egger’s test and Begg’s test showed no evidence of publication bias for the included studies on each parameter, which was showed in Table 2. The funnel plots were showed in S1 Fig.

**Table 2:** The results of publication bias.

Parameters	Egger’s test		Begg’s test	
	*t* value	*p* value	*z* value	*p* value
CoQ10	1.00	0.50	1.57	0.12
IL-6	-1.43	0.29	-0.68	0.50
TNF-α	-0.83	0.56	-0.52	0.60
CRP	-0.75	0.49	-0.75	0.45

### 3.7 Side-effects

Five of nine trials provided information on side-effects[17,18,20,22,23]. Among them, only one trial reported that 2 patients from the CoQ10 group has the minor adverse effects of redness and itchiness of the skin during the study[18].

## 4. Discussions

In this systematic review and meta-analysis, we summarized published evidence from nine RCTs that investigated the effects of CoQ10 supplementation on systematic inflammation markers as measured by pro-inflammatory factors (IL-6, TNF-α, CRP). The main finding of the review was that the serum level of CoQ10 were significantly improved and TNF-α level was significantly decreased in CoQ10 supplementation group compared with placebo group in patients with metabolic diseases. However, CoQ10 supplementation did not significantly decreased CRP and IL-6 level.

Adipocytokines are a variety of bioactive molecules (including IL-6, TNF-α) produced and secreted by adipose and other tissues[24]. CRP is a sensitive and dynamic systemic marker of inflammation synthesized in the liver[25]. Increased level of IL-6, TNF-α and CRP are thought to contribute to the development of insulin resistance, T2DM, and CVD[24]. CoQ10, a well-accepted nutritional supplement and anti-oxidant agent, was known to play a protective role in various physiological and pathological processes. So far several studies have identified the anti-inflammatory function of CoQ10[23,26]. However, it remains unclear what is their mechanism of action. Several potential mechanisms might partially explain it. It seems that CoQ10 might play a potential role in decreasing the production of pro-inflammatory cytokines by inhibiting NF-κB gene expression[27], attenuating miR-146a and IL-1 receptor associated kinase modulation[28] and reducing the secretion of macrophage inflammatory protein-1 alpha and regulated upon activation normal T-cell expressed and secreted factors[29]. In addition, several studies observed a negative relationship between adiponectin and inflammatory factors (TNF-α, IL-6, hs-CRP) [30,31]. Zhou et.al reported adiponectin can inhibit stimulated monocytes secreting TNF-α [31]. So it is likely that rise in adiponectin by using of coenzyme Q10[18,23] can indirectly bring about a plunge in inflammatory factors such as TNF-α.

No effect of CoQ10 supplementation on CRP and IL-6 was found in this systematic review. The associations of CRP, IL-6 and CoQ10 serum concentration were influenced by age, sex, BMI, lipoprotein concentration and health status [32,33]. Although the sources of heterogeneity was explored with none covariates influenced the pooled effect, the impact of different conditions with none measured parameters from the subjects on the effect of CoQ10 intervention should not be ruled out. Moreover, the short intervention period, the different dose for intervention and the limited number of participants enrolled in the RCTs may have contributed to the observed null effect of CoQ10 on the serum level of CRP and IL-6.

Several limitations should be acknowledged. Firstly, the present meta-analysis focused only on papers published in English, the ones that reported in other languages may affect the present results. Secondly, most of subjects included in our meta-analysis (eight of nine studies) came from Iran, China and Korea. So the results may be only applicable to the Asian population. Thirdly, although evidence from Egger’s and Begg’s test suggested that publication bias was unlikely, the number of included studies was somewhat small. Finally, because of the limited number of included RCTs, the subgroup analyses were not conducted in this paper. Further rigorously designed RCTs with larger sample sizes and long enough duration are needed to confirm the effectiveness of CoQ10 supplementation for specified metabolic diseases.

## 5. Conclusion

The present systematic review provides some evidence that CoQ10 supplementation may partly improve the process of inflammatory state in patients with metabolic diseases. However, the results should be interpreted with caution because of the evidence of heterogeneity. Further studies, especially with larger sample size and well-designed RCTs, are needed to confirm the effectiveness of CoQ10 supplementation on benefitting to inflammation status in metabolic diseases.

## Supporting Information

S1 Checklist PRISMA 2009 checklist. (DOC)

S1 Fig Funnel plot for publication bias of Coenzyme Q10 supplementation and serum lipids (outcomes: A: serum Coenzyme Q10, B: tumor necrosis factor-alpha, C: interleukin-6, D: C reactive protein). (TIF)
