# Supplementary figures and images for: Effects of Coenzyme Q10 on Markers of Inflammation: A Systematic Review and Meta-Analysis
Source: PLoS One. 2017 Jan 26;12(1):e0170172. doi: 10.1371/journal.pone.0170172 (PMC5268485; doi:10.1371/journal.pone.0170172)

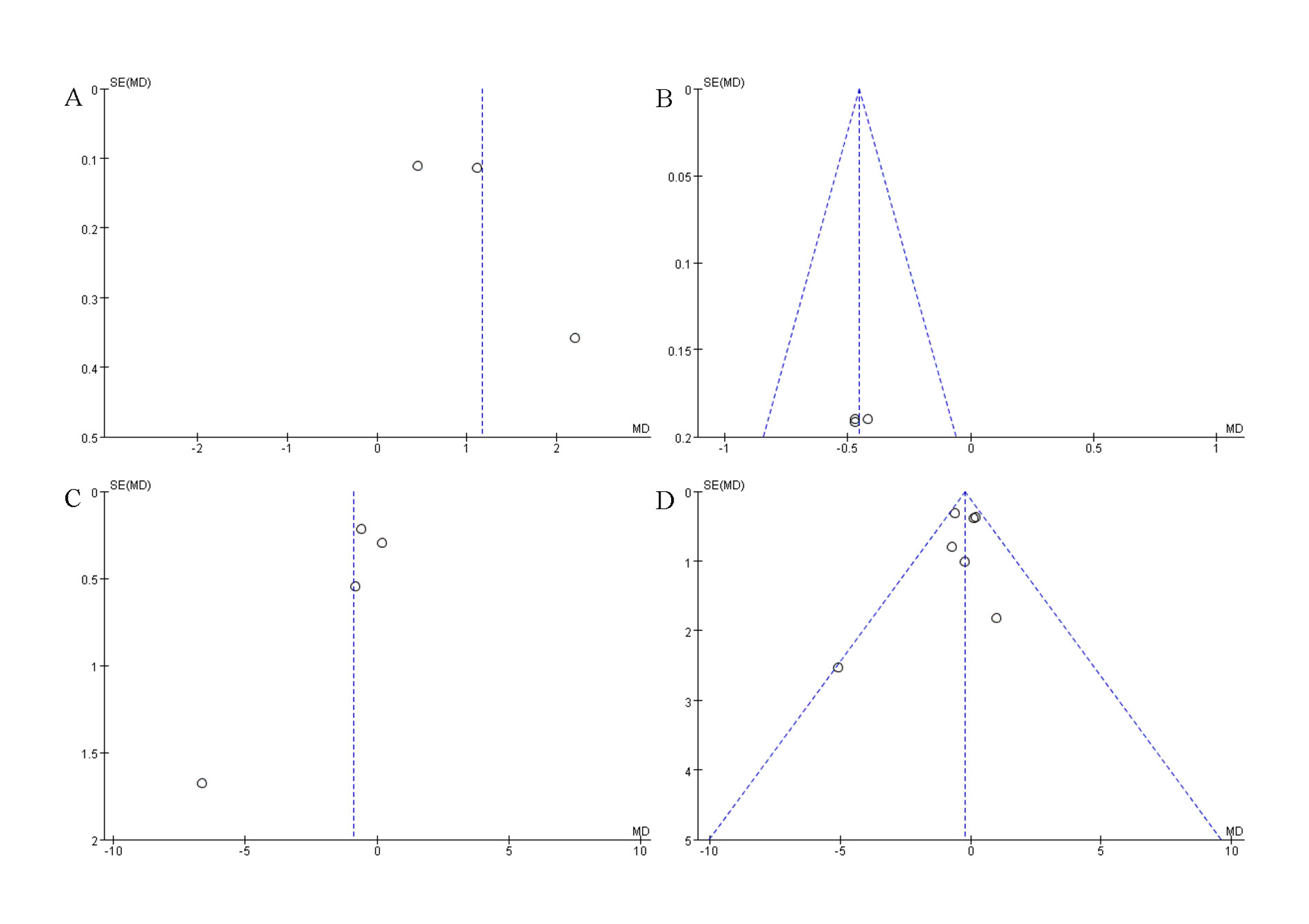

Supplement: S1 Fig — (TIF) [file pone.0170172.s002.tif]
